# Drying or anaerobic digestion of fish sludge: Nitrogen fertilisation effects and logistics

**DOI:** 10.1007/s13280-017-0927-5

**Published:** 2017-06-07

**Authors:** Eva Brod, Johan Oppen, Annbjørg Øverli Kristoffersen, Trond Knapp Haraldsen, Tore Krogstad

**Affiliations:** 1 NIBIO, Norwegian Institute of Bioeconomy Research, P.O. Box 115, 1431 Ås, Norway; 2 Møreforsking Molde AS, Britvegen 4, 6410 Molde, Norway; 3 Faculty of Environmental Sciences and Natural Resource Management, Norwegian University of Life Sciences, P.O. Box 5003, 1433 Ås, Norway

**Keywords:** Aquaculture, Fish farming, Plant availability, Plant nutrition, Recycling, Waste

## Abstract

Application of fish sludge as fertiliser to agricultural land can contribute to closing nutrient cycles in fish farming. The effect of different treatment technologies on the nitrogen fertilisation effects of fish sludge was studied by a bioassay with barley (*Hordeum vulgare*), an incubation and a field experiment. Dried fish sludge resulted in relative agronomic efficiency of 50–80% compared with mineral fertiliser. The anaerobic digestate based on fish sludge (20 vol%) and dairy manure did not increase nitrogen uptake in barley. Increasing the ratio of fish sludge in the digestate increased the fertilisation effect, but requires optimisation of the biogas process. A simple logistics analysis conducted for a case hatchery showed that on-site drying and co-digestion of fish sludge in a central biogas plant can be regarded as equal in terms of costs. Norway can become an exporter of fish sludge-based recycling fertilisers if current regulations are modified to facilitate nutrient recycling.

**Electronic supplementary material:**

The online version of this article (doi:10.1007/s13280-017-0927-5) contains supplementary material, which is available to authorized users.

## Introduction

Optimal utilisation of nutrients in waste resources will be crucial in the future circular economy. Substituting mineral fertiliser with waste resources can contribute to closing nutrient cycles and to coping with challenges related to depletion of finite resources. In Norway, around 27 000 Mg of nitrogen (N) and 9000 Mg of phosphorus (P) are lost to the sea as feed residues and faeces (fish sludge) during fish farming each year (Hamilton et al. 2016). Nutrient losses with fish sludge are in the same order of magnitude as nutrients in animal manure. At the same time, today’s aquaculture production is dependent on the import of feed ingredients, e.g. fishmeal and soya. The linearity of nutrient flows makes current fish farming practices highly unsustainable. Norway was the largest per capita aquaculture producer in the world in 2013 (FAO 2013), with salmon accounting for the largest share of fish exports. Norwegian aquaculture production has been anticipated to increase fivefold from 2010 to 2050 (DKNVS and NTVA 2012), implying a fivefold increase in nutrient imports and nutrient losses. Production growth can only be sustainable if fish sludge is recognised as a valuable resource of nutrients and collected to be utilised, e.g. by substituting for mineral fertiliser in Norway or abroad.

Currently, only new on-land hatcheries and those expanding their capacity are obliged to collect fish sludge before discharging water to the sea (Norwegian Ministry of Climate and Environment 2004). It is common practice to apply this untreated fish sludge from hatcheries to agricultural land together with manure. However, the amount of fish sludge requiring treatment is expected to increase due to the trend for increasing smolt lifetime in on-shore systems (≤1 kg living weight). Increasing amounts of fish sludge will require new solutions for handling continuously increasing amounts of fish sludge. Different treatment technologies are currently under development. Treatments include mechanical filtering before application of drying techniques that can increase the dry matter (DM) content of fish sludge to up to 90%. Dried fish sludge can then be pelleted to produce recycling fertilisers, which can be spread on agricultural land with conventional fertiliser spreaders. Anaerobic treatment of fish sludge, with or without co-digestion of manure, has also gained attention, as the organic carbon (C) it contains is transformed into biogas. The nutrients remain in the liquid digestate, which is characterised by low DM content. Digestate can be spread to agricultural land by equipment designed for application of cattle or pig slurry.

The interest in developing new technologies for fish sludge handling has in the past been influenced by keeping costs low, rather than by their effect on plant availability of nutrients in the residual product. The effect of dried fish sludge applied as N fertiliser to ryegrass or spring cereals has been shown to be as high as 50–90% of the effect of mineral fertiliser during pot experiments with nutrient-deficient soils (Brod et al. 2012, 2014). In pot experiments with nutrient-deficient soils, the effect of dried fish sludge as P fertiliser to ryegrass or barley has been shown to be comparable to that of dairy manure (Brod et al. 2015, 2016). However, the effect of different treatment technologies on the N fertilisation effect of fish sludge has not been studied previously.

The aim of this study was thus to evaluate the N fertilisation effects of the two main treatment technologies for fish sludge: drying and anaerobic digestion. During a bioassay with barley (*Hordeum vulgare* var. Heder), the N fertilisation effects of seven fish sludge-based recycling fertilisers were compared with those of well-known N-rich recycling fertilisers and mineral N fertiliser, as well as an unfertilised control treatment. In parallel, soil-fertiliser incubations were conducted to compare N mineralisation rates of the recycling fertilisers with plant N uptake. Further, a field experiment was conducted to compare the N fertilisation effect of dried fish sludge with that of mineral N fertiliser. The results of the growth and incubation experiments are discussed in light of the outcome of a simple logistics analysis performed to evaluate costs related to two fish sludge treatment alternatives at a case hatchery.

## Materials and methods

### Fish sludge and recycling fertilisers used in the bioassay and incubation experiment

Fish sludge products and reference recycling fertilisers used in the bioassay and incubation experiment are described in Table 1, while Table 2 provides an overview of selected chemical properties. The DM content was determined after drying at 105 °C and organic matter (OM) content was quantified after incineration at 550 °C. Total organic carbon (TOC) was determined by incineration at 105 °C and simultaneous measurement of CO_2_ by an infrared measuring cell following washing with 3% HCl, or according to EN 15936 (2012). The pH was determined on sieved samples in deionised H_2_O in a solid:solution ratio 1:5 (v/v) or directly in the liquid samples (fish sludge, digestates and dairy manure). Total N content was determined by the modified Kjeldahl method (EN 13654-1 2001), while nitrate-N (NO_3_-N) and ammonium-N (NH_4_-N) were determined after extraction in deionised water or directly in the liquid samples. Total P, potassium (K), magnesium (Mg), calcium (Ca), sulphur (S) and heavy metal content were determined by ICP-MS after digestion in concentrated nitric acid in an ultraclave or autoclave (EN ISO 11885 2009).

**Table 1:** Description of fish sludge-based recycling fertilisers and reference recycling fertilisers

Product	Description
Fish sludge	Collected from the on-land Marine Harvest Haukå salmon hatchery, Norway. The effluent containing faeces and feed residues was mechanically filtered through a Salsnes filter
Fish sludge digestate 1	Digestate obtained after anaerobic treatment of dairy manure and fish sludge in two continuous batch processes (13 L per reactor) at the NIBIO station at Tingvoll. The reactors were fed fresh substrate daily and the equivalent amount was removed. After stabilising the biogas process on 600 mL manure per day, the reactors were fed 150 mL pure fish sludge. The percentage of fish sludge in the reactors reached a peak of 28 vol% after 2 days, after which only manure was added to allow the bacteria to break down accumulated volatile fatty acids. At the conclusion of the experiment, the fraction of fish sludge in the reactor was 13 vol%. The digestate used here was a blend of samples collected over 1.5 months and contained approximately 20 vol% fish sludge
Fish sludge digestate 2	Digestate after anaerobic treatment of dairy manure and fish sludge in two continuous batch processes, comparable to the fish sludge digestate 1. However, in this case the two reactors were fed a mixture of 20 vol% fish sludge (75 mL) and 80 vol% manure (300 mL). At the conclusion of the experiment, the percentage of fish sludge in the reactor was 14 vol%. The digestate used here was a blend of samples collected over 1.5 months and contained approximately 11 vol% fish sludge
Fish sludge digestate 3	Digestate after anaerobic treatment of dairy manure and fish sludge in two continuous batch processes. Two reactors were started with 8.6 L digested manure from the biogas plant at NIBIO Tingvoll and operated for 130 days with addition of variable amounts of fish sludge 5 days per week. No sludge was removed. At sampling, the percentage of fish sludge in the reactor was 33 vol%
Fish sludge digestate 4	Digestate after anaerobic treatment of dairy manure and fish sludge in two continuous batch processes, comparable to fish sludge digestate 3. However, in this case 70 mL fish sludge were added 5 days per week. At sampling, the percentage of fish sludge in the reactor was 42 vol%
Fish sludge pellets	Collected from the on-land Sævareid salmon hatchery after mechanical filtering before treatment in a reactor developed by the company Global Enviro. The material was then pelleted at the Norwegian Paper and Fibre Institute, Trondheim
Fish sludge granules	Collected from the on-land Flatanger settefisk salmon hatchery after sedimentation and flocculation following addition of a polymer. The fish sludge was then dried on a belt dryer developed by Sterner Aquatek AS
Meat-bone meal pellets	Stabilised and sanitised slaughterhouse waste from the slaughterhouse in Mosvik. Pelleted at the Norwegian Paper and Fibre Institute
Food waste pellets	Source-separated catering waste from Rica Sunnfjord Hotel aerobically treated and dried in a reactor developed by the company Global Enviro after separation of grease and water by steam and pressure. Pelleted at the Norwegian Paper and Fibre Institute
Lindum food waste	Source-separated municipal organic waste mixed with 2–5% hydrated lime before aerobic treatment in a reactor developed by Lindum Bioplan at 50–70 °C for 15–21 days. Material was screened at 10 mm to remove impurities
Paper mill sludge pellets	A mixture of fibre sludge and biological sludge from the pulp and paper plant Fiborgtangen, Norske Skog, Skogn. The material was dried and pelleted at the Norwegian Paper and Fibre Research institute
Dairy manure	Slurry from organic dairy cows, collected from the dairy house at NIBIO, Tingvoll after being pumped out through a Rotacut 3000, which has rotating blades that reduce particle size and homogenise the slurry
Chicken manure	Stabilised, sanitised and pelleted chicken manure produced and marketed by Norsk Naturgjødsel

**Table 2:** Selected chemical properties of fish sludge-based recycling fertilisers and organic reference fertilisers

		Fish sludge	Fish sludge digestate 1	Fish sludge digestate 2	Fish sludge digestate 3	Fish sludge digestate 4	Fish sludge pellets	Fish sludge granules	Meat-bone meal pellets	Food waste pellets	Lindum food waste	Paper mill sludge pellets	Dairy manure	Chicken manure
DM	g 100 g^−1^	13	3.8	3.7	3.9	4.9	100	95.2	100	100	78.8	100	6.1	93.4
OM	g 100 g^−1^ DM	79	67	68	62	65	91	88	81	89	63	84	79	–
TOC	g 100 g^−1^ DM	23	25	28	32	30	51	–	46	50	35	43	24	–
pH		5.8	8.4	8.4	8.4	8.3	5.7	5.5	5.9	5.6	8.0	6.5	8.2	6.14
N	kg Mg^−1^	11	8	7	3	6	75	68	102	41	23	24	3	60
N	g kg^−1^ DM	82	220	190	87	130	75	71	102	41	29	24	50	65
C/N		2.8	1.1	1.5	3.7	2.3	6.8	–	4.5	12.3	12.1	17.8	4.8	5.1
NH_4_-N	g kg^−1^ DM	6.9	60	44	100	110	1.7	0.5	0.33	0.46	1.5	0.95	19	5
NO_3_-N	g kg^−1^ DM	0.027	0.05	0.017	0.0076	n.d.	0.0026	0.00117	0.00143	0.043	n.d.	0.0061	0.048	–
Nmin	% of total N	8.4	27.3	23.2	115.0	84.6	2.3	0.7	0.3	1.2	5.2	4.0	38.1	7.7
P	g kg^−1^ DM	24	26	17	26	31	15	14	33	4.7	3.8	4.6	10	37
K	g kg^−1^ DM	8.2	150	200	47	46	1.9	0.27	4.1	7.7	5.5	0.85	66	47
S	g kg^−1^ DM	8.4	13	9.8	7.9	9.6	5	5.4	5.9	2.8	2.4	4.3	6.1	24
Ca	g kg^−1^ DM	42	57	2.8	53	63	21	28	62	23	57	32	24	77
Mg	g kg^−1^ DM	5.7	10	11	4.2	3.8	2.1	1.4	1.5	1.2	1.3	1.1	9.3	2.1
Al	mg kg^−1^ DM	43	650	500	400	460	140	230	110	140	650	4800	440	–
Fe	mg kg^−1^ DM	690	1900	1300	1400	1700	400	790	420	260	1900	110	870	410
Cd	mg kg^−1^ DM	0.77^I^	1.4^II^	0.69^I^	1.1^II^	1.7^II^	0.47^I^	0.26^0^	0.015^0^	0.036^0^	0.054^0^	0.32^0^	0.015^0^	0.17^0^
Pb	mg kg^−1^ DM	0.59^0^	1.8^0^	1.5^0^	0.77^0^	0.76^0^	0.27^0^	0.17^0^	0.13^0^	0.36^0^	0.24^0^	6.5^0^	–	0.94^0^
Hg	mg kg^−1^ DM	0.038^0^	0.121^0^	0.059^0^	0.098^0^	0.141^0^	0.041^0^	0.038^0^	n.d.^0^	0.03^0^	–	0.039^0^	–	0.008^0^
Ni	mg kg^−1^ DM	1.2^0^	13^0^	14^0^	12^0^	15^0^	0.8^0^	0.6^0^	0.98^0^	2.2^0^	1.2^0^	8.1^0^	–	5.3^0^
Zn	mg kg^−1^ DM	410^II^	800^II^	430^II^	750^II^	990^III^	190^II^	430^II^	84^0^	33^0^	110^0^	76^0^	84^0^	180^I^
Cu	mg kg^−1^ DM	22^0^	90^I^	67^I^	57^I^	68^I^	13^0^	17^0^	5.4^0^	8.2^0^	16^0^	43^0^	5.4^0^	44^0^
Cr	mg kg^−1^ DM	4.8^0^	18^0^	15^0^	18^0^	22^0^	1.9^0^	4.2^0^	2.6^0^	5.6^0^	1.8^0^	12^0^	–	3^0^

*n.d.* not detectable. For parameter abbreviations, see text

Superscripts on heavy metal concentrations denote quality class 0, I, II, III according to the Norwegian classification (Norwegian Ministry of Agriculture and Food 2003)

### Bioassay experiment

The bioassay was conducted in a greenhouse with 5-L pots filled with a nutrient-deficient 9:1 (v/v) blend of sand and moist sphagnum peat (7.53 kg pot^−1^ with 1.51 kg DM L^−1^, pH 7, <2.0 mg P–AL 100 g^−1^, <2.0 mg K–AL 100 g^−1^, 2.5 mg Mg–AL 100 g^−1^ and 24 mg Ca–AL 100 g^−1^ where AL = extraction with 0.1 M ammonium lactate and 0.4 acetic acid adjusted to pH 3.75 according to Egnér et al. (1960) and analysis on ICP-OES). This model soil was chosen to prevent N fertilisation effects of fertiliser treatments being masked by the soil. Fertilisation rates for the recycling fertilisers were calculated based on total N content, equivalent to 200 and 400 mg N pot^−1^ (26.5 and 53.1 mg N kg^−1^ soil and 80 and 160 kg N ha^−1^, respectively, assuming 20 cm topsoil depth). Fish sludge products and recycling fertiliser were mixed into the upper 5 cm of the soil, imitating harrowing. Their fertilisation effects were compared with a treatment receiving no N fertiliser (NoN) and mineral control treatments (MinN) receiving Ca(NO_3_)_2_ equivalent to 200 and 400 mg N pot^−1^. There were three replicates per treatment. All pots received an N-free nutrient solution containing 7 mg P kg^−1^ soil and 27 mg K kg^−1^ soil when 200 mg N pot^−1^ was applied, or 13 mg P kg^−1^ soil and 53 mg K kg^−1^ soil when 400 mg N pot^−1^ was applied. All pots also received 8.2 mg Mg kg^−1^ soil, 1 mg iron (Fe) kg^−1^ soil, 0.01 mg molybdenum (Mo) kg^−1^ soil, 2 mg manganese (Mn) kg^−1^ soil, 3.1 mg copper (Cu) kg^−1^ soil, 0.02 mg boron (B) kg^−1^ soil and 1.1 mg zinc (Zn) kg^−1^ soil. Twenty seeds of barley (*Hordeum vulgare*, var. Heder) were sown per pot and thinned out to 15 plants per pot after germination. All pots were watered by weighing to 60% of water-holding capacity (WHC, 100% WHC = 214 g H_2_O kg^−1^ soil determined by free draining after water saturation for 24 h) every two or three days and daily towards the end of the experiment. Growing conditions in the greenhouse were set to 16 h photoperiod, with artificial lights turning on when daylight was <300 W m^−2^. The mean temperature was set to 20 °C during the day and 16 °C at night. Pot positions were randomised each time the plants were watered. When all barley plants were ripe, approximately 12 weeks after sowing, they were cut manually with scissors at a height of 5 cm above the soil surface. The harvested biomass was threshed and the straw and grain fractions were weighed separately before milling. Total N in straw and grain was determined by the Dumas method (EN 13654-2 2001), while the concentration of total P, K, Mg, Ca and micronutrients was determined as described for the recycling fertilisers. Nitrogen uptake in straw and grain was computed by multiplying N concentration by DM production.

To evaluate N fertilisation effects in the bioassay, apparent nitrogen recovery (ANR, %) in total aboveground biomass was calculated based on the difference method as

1 $$ {\text{ANR }}\left( \% \right) = \frac{{{\text{N uptake }}\left( {{\text{N}}+} \right) - {\text{N uptake }}({\text{NoN}})}}{\text{N applied}}, $$

where N uptake (N+) (mg N pot^−1^) is N taken up in aboveground biomass by fertilised plants, N uptake (NoN) (mg N pot^−1^) is N taken up in aboveground biomass by the average of NoN plants and N applied (mg N pot^−1^) is N applied with the fertiliser (mg N pot^−1^).

**Fig. 1:**
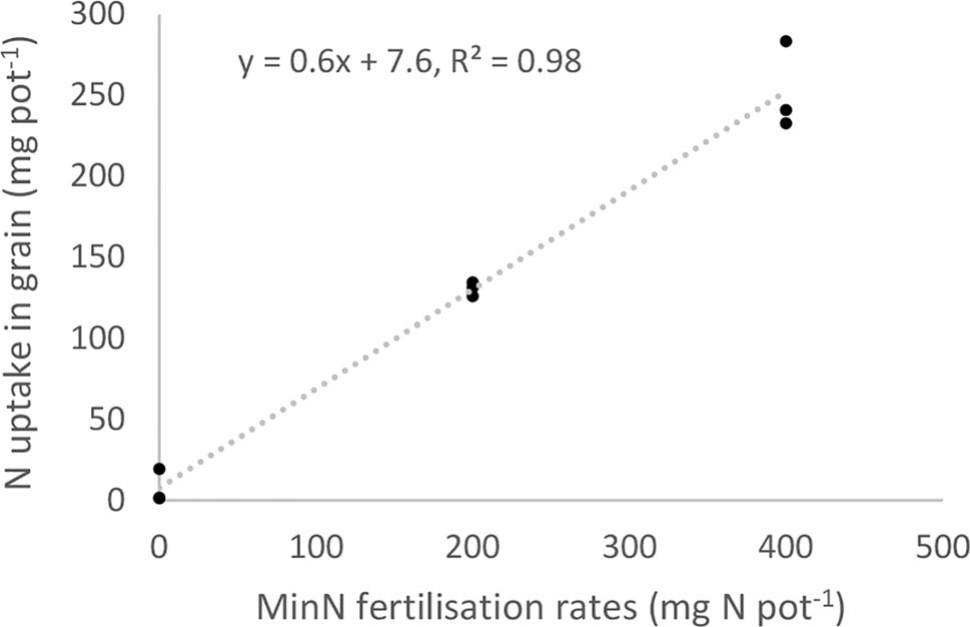
Nitrogen (N) uptake in grain (mg N pot^−1^) as an effect of increasing mineral nitrogen (MinN fertilisation rate (0, 200 and 400 mg N pot^−1^) in the bioassay. Example of calculation of relative agronomic efficiency (RAE): Y_1_ = 19.25 g grain biomass pot^−1^ and X_1_ = 19.4 mg N pot^−1^ were the values used for calculation of RAE = 10% of replicate 1 of dairy manure (200 mg N pot^−1^)

To compare the fertilisation effects of fish sludge products and recycling fertilisers with those of MinN in the bioassay, their relative agronomic efficiency (RAE, %) was calculated for grain yield according to

2 $$ {\text{RAE}} = 100 \times \frac{{{\text{X}}_{1} }}{\text{N applied}} $$

and

3 $$ {\text{X}}_{1} = \frac{{({\text{Y}}_{1} - {\text{b}})}}{\text{a}}, $$

where Y_1_ is grain yield obtained after application of fish sludge or recycling fertiliser, X_1_ is amount of MinN (mg N pot^−1^) to which Y_1_ is equivalent, and a and b are the slope and intercept obtained from linear regression with Y being grain yield (g pot^−1^) obtained after application of MinN and X being N application rate of MinN (0, 200 and 400 mg N pot^−1^). For the parameters a and b and an example of how RAE is calculated, see Fig. 1.

### Incubation experiment

Soil-fertiliser incubations were conducted to study N mineralisation in fish sludge and recycling fertilisers over time, using the same model soil as in the bioassay. Basically, the same fertiliser treatments were used as in the bioassay. However, dairy manure was not studied in the incubation experiment, but Lindum food waste (see Table 1) was included. During set-up of the incubation experiment, 500 g portions of soil were watered to 60% of WHC and mixed with the fertiliser treatments equivalent to the highest rate in the bioassay at a rate corresponding to 400 mg N pot^−1^ soil. To study mineral N in three replicates at six time points (1, 3, 7, 14, 32 and 64 days), 18 sub-portions of 20 g DM were prepared for each treatment in 100-mL sealable glass jars. The jars were kept in a climate chamber at 16 °C and opened once a week to keep the environment aerobic. At sampling, 40 mL 2 M KCl were added to each sub-aliquot and NO_3_-N and NH_4_-N were extracted for 1 h. Extracts were stored in a refrigerator before analysis using a Konelab Aqua 60 analyser. For comparison of mineral N in the soil to the effect of the different fertiliser treatments, mineral N in the NoN control soil was subtracted at all time points (0.9, 1.3, 1.1, 1.2, 0.7 and 0.3 mg N kg^−1^ soil after 1, 3, 7, 14, 32 and 64 days).

### Field experiment

The field experiment was conducted at two sites: Apelsvoll (60°42′N, 10°51′E) situated in eastern inland Norway, which has a drier climate and lower winter temperature than Værnes (63°27′N, 10°57′E) in central Norway, which has a humid coastal climate. Normal annual precipitation is 600 mm in Apelsvoll and 896 mm in Værnes (average for the period 1961–1990), respectively, half of which occurs during the period May–September. The normal mean air temperature in the growing season is 12–13 °C at both sites. The soil at Apelsvoll is a sandy loam (14% clay and 55% sand) classified as an Endostagnic Cambisol. The soil at Værnes is a sandy loam (6% clay and 51% sand) classified as an Arenic Fluvisol (IUSS Working Group WRB 2006). The experiment was designed as a randomised block design with three replicates and different sites at Apelsvoll during 2012 and 2013. At both Apelsvoll and Værnes, the crop grown before the experiment was cereal. The soil was ploughed in autumn 2011 and harrowed in spring 2012 before set-up of the experiment. Dried fish sludge, food waste and meat-bone meal combined with anaerobic digestate based on source-separated food waste were applied by hand. Chemical properties of the recycling fertilisers used in the field experiment are presented in the 10.1007/s13280-017-0927-5. Mineral N fertiliser (NPK compound fertiliser 22-3-10) was applied during sowing at Apelsvoll and spread by hand at Værnes. The intention was to apply all fertilisers based on 80 kg total N ha^−1^, but actual fertilisation rates deviated partly from this. The treatments were not supplemented by other fertilisers. The soil was again harrowed after application of the fertiliser. Sowing was carried out diagonally to the direction of fertiliser application. The soil was rolled after sowing and weeded once or twice with a tined weeder. At Apelsvoll, both barley (*Hordeum vulgare* var. Brage) and wheat (*Triticum aestivum* var. Bjarne) were sown each year, while at Værnes only barley was sown. The plots were not treated against diseases or pests. Grain yield was measured on a 1.5 m × 5–6 m sub-area within each plot. Grain protein content was measured by near infrared reflectometry (INFRA 250, TECnicon, US). The N concentration in grain was calculated by dividing crude protein by a factor of 6.25 for barley and 5.75 for wheat. Nitrogen uptake in grain was computed by multiplying N concentration by DM production. Apparent nitrogen recovery in grain (%) was calculated based on the difference method as described for the bioassay. Relative agronomic efficiency in the field was calculated for N uptake in grain according to

4 $$ {\text{RAE}}_{\text{field}} = \frac{{{\text{ANR }}({\text{N}}+)}}{{{\text{ANR }}({\text{MinN}})}} \times 100, $$

where ANR (N+) is ANR in grain of fertilised plants and ANR (MinN) is average ANR in grain of plants receiving MinNPK.

Results are only presented for those years in which fish sludge was applied.

### Analysis of experimental data

To analyse the data obtained in the bioassay, two-way ANOVA was applied to test the effect of the factors fertiliser treatment and fertilisation rate and their interaction on different parameters. Data sets were also analysed using one-way ANOVA within each fertilisation rate, and data from the incubation and field experiment were analysed using one-way ANOVA. Analysed data were checked for normal distribution (normal quantile plots) and homogeneity of variance (residual vs fitted plots). For pair-wise comparisons, Tukey’s honestly significant difference (HSD) test was used at significance level *α* = 0.05. Moreover, simple linear regressions were run with averaged replicates of RAE of the bioassay as response variable and selected chemical properties and parameters of the incubation experiment as explanatory variables.

### Logistics

A simple logistics analysis was conducted using the hatchery Smøla Klekkeri og Settefiskanlegg AS, located in Smøla in Møre and Romsdal county, on the north-west coast of Norway as a representative case for Norwegian smolt hatcheries (Oppen and Oterhals 2016). At this hatchery, yearly production of 2.5 million smolt results in approximately 665 Mg of fish sludge with 10% DM. Rapid changes and development of new technology in the industry make it difficult to get good cost estimates, and no reliable sources for such estimates could be found in the literature. Calculations were therefore based on qualified guesses from experts, and cost estimates are uncertain. Costs related to transportation and handling of all sludge produced yearly were estimated in Norwegian kroner (NOK) before conversion to Euros (€), assuming an exchange rate of 9 NOK to 1 €.

For the *Fish sludge digestate* treatment, the fish sludge was assumed to be transported by tank truck to a centralised biogas plant for further treatment. What happens after the sludge is delivered, including transportation of digestate to agricultural land, was not considered. For this alternative, the hatchery would need to invest in a tank where sludge is stored before it is transported to the plant. We assumed there is no need for extra labour to store the sludge. The capital investment was estimated to be €28,000 (Martinsen, pers. comm.), with a depreciation period of 20 years, while the transportation costs were estimated to be €1300 per truckload of 22 Mg of sludge (Martinsen, pers. comm.). The gate fee at the biogas plant was estimated to be €0.09 per kg sludge delivered in 2017 (Ecopro, pers. comm.).

For the *Dried fish sludge* alternative, the sludge was assumed to be dewatered, dried and pelleted at the hatchery before being packed in bags and sent to eastern Norway to be sold as fertiliser. We assumed that equipment for dewatering and drying the sludge to 90% DM, followed by pelleting, requires an investment of €350,000 (Martinsen, pers. comm.) with a depreciation period of eight years and a yearly energy cost of €13,000. We also assumed that half a person-year of labour is needed to run these processes, accounting for costs of €50,000 (Martinsen, pers. comm.). Transportation of 74 Mg of sludge pellets to eastern Norway was expected to cost €4440. As a sales price, we assumed €0.35 per kg sludge pellets (Felleskjøpet 2014), giving a total revenue of €25,900. Financial costs were calculated based on an annual interest rate of 5% for both alternatives.

## Results

### Total and mineral N in recycling fertilisers studied in the bioassay and incubation experiment

In total, seven different fish sludge-based recycling fertilisers were studied in the bioassay. These were five liquid products (untreated fish sludge, four anaerobic digestates (1–4) based on co-digestion of fish sludge and dairy manure) and two dry products (pellets and granules). The fish sludge-based recycling fertilisers contained 71–220 g N kg^−1^ DM, with the lowest content in fish sludge granules and the highest N content in digestate 1 (Table 2). Untreated fish sludge contained 82 g N kg^−1^ DM. The fraction of mineral N in fish sludge-based recycling fertilisers varied considerably. In untreated fish sludge, 8% of total N was present as mineral N. Digestates 1 and 2 contained 20–30% of total N as mineral N, while in digestates 3 and 4 >80% of total N was present as mineral N. In digestate 3, the NH_4_-N concentration exceeded the concentration of total N, indicating uncertainty in the analytical methods. The dry fish sludge pellets and granules contained only 0.7–2.3% of total N as mineral N. In the reference recycling fertilisers, the N content ranged between 24 and 102 g N kg^−1^ DM, with the lowest content in paper mill sludge pellets and the highest in meat-bone meal, and mineral N fractions were low for all reference recycling fertilisers.

In Norway, the use of recycling fertilisers is regulated by the concentration of heavy metals per unit DM, based on classification into three quality classes (Norwegian Ministry of Agriculture and Food 2003). All fish sludge-based recycling fertilisers were in quality class II due to elevated Zn and/or Cd level (Table 2). Products in quality class II may be applied to agricultural land at a rate of up to 20 Mg DM per hectare and 10-year period. Digestate 4 was in quality class III due to elevated Zn concentration and therefore may not be applied to agricultural land. The reference recycling fertilisers were in quality class 0 and may be applied to agricultural land at rates not exceeding crop requirements, except for chicken manure which was in quality class I, limiting its use to 40 Mg DM per hectare and 10-year period.

### Nitrogen fertilisation effects of recycling fertilisers in the bioassay

There was a clear response of barley to N application on the experimental soil, as shown by the linear increase in N uptake in grain as a function of increasing MinN application rate (0, 200 and 400 mg N pot^−1^) (Fig. 1). The N concentration in grain ranged from 0.81 to 1.09 g 100 g^−1^ DM (Table 3) and was clearly below critical levels for all fertiliser treatments, indicating N limitation (DTU Fødevareinstituttet 2016). All other nutrient concentrations in grain were within the ranges indicating sufficient supply (results not shown), and observed differences between fertiliser treatments were therefore ascribed to N fertilisation effects.

**Table 3:** Grain and straw biomass production, nitrogen (N) concentration in grain and straw, apparent nitrogen recovery (ANR) and relative agronomic efficiency (RAE) as an effect of different fertiliser treatments at two fertilisation rates (200 and 400 mg N pot^−1^) in the bioassay

Treatment	Biomass		N concentration		ANR	RAE
	Grain	Straw	Grain	Straw		
	g pot^−1^	g pot^−1^	g 100 g^−1^ DM	g 100 g^−1^ DM	%	%
200 mg N pot^−1^						
Control	0.8	1.5	0.92	0.38	–	–
MinN	14.8	13.3	0.88	0.35	82	100^a^
Fish sludge	8.5	6.3	0.86	0.22	37	57
Fish sludge digestate 1	2.2	3.1	0.90	0.24	7	8
Fish sludge digestate 2	1.6	2.8	0.97	0.19	4	3
Fish sludge digestate 3	14.2	10.2	0.88	0.22	67	101
Fish sludge digestate 4	9.5	7.4	0.84	0.15	38	65
Fish sludge pellets	9.6	6.7	0.92	0.17	43	65
Fish sludge granules	9.1	6.9	0.89	0.18	40	62
Meat-bone meal pellets	9.3	6.9	0.88	0.16	40	63
Food waste pellets	6.0	4.2	1.09	0.20	30	37
Paper mill sludge pellets	4.8	4.1	0.94	0.16	19	28
Dairy manure	2.8	3.2	0.95	0.24	11	13
Chicken manure	8.2	6.4	0.83	0.20	34	55
HSD	1.9	1.1	0.14	0.13	8	14
SEM	0.4	0.2	0.26	0.03	2	3
400 mg N pot^−1^						
Control	0.8	1.5	0.92	0.38	–	–
MinN	26.6	19.0	0.95	0.33	75	100^a^
Fish sludge	18.9	12.7	0.93	0.32	51	69
Fish sludge digestate 1	6.4	5.9	0.81	0.28	14	20
Fish sludge digestate 2	3.2	4.7	0.95	0.34	8	0
Fish sludge digestate 3	24.5	17.5	0.94	0.37	71	90
Fish sludge digestate 4	17.0	13.0	0.90	0.26	43	62
Fish sludge pellets	18.9	12.6	0.94	0.27	49	69
Fish sludge granules	17.0	11.7	0.94	0.27	45	61
Meat-bone meal pellets	15.7	10.5	0.94	0.18	39	56
Food waste pellets	11.4	7.0	1.07	0.12	29	40
Paper mill sludge pellets	10.0	6.8	0.94	0.15	23	34
Dairy manure	6.1	6.2	0.90	0.17	13	19
Chicken manure	14.9	11.0	0.92	0.19	36	53
HSD	2.2	1.7	0.16	0.17	10	7
SEM	0.4	0.3	0.03	0.03	2	1
Two-way ANOVA, source of variation						
Treatment	***	***	***	***	***	***
Fertilisation rate	***	***	n.s.	***	***	*
Treatment × fertilisation rate	***	***	n.s.	**	***	***

*n.s.* not significant

^a^By definition set to 100%. SEM = pooled standard error of the mean and HSD = Tukey’s honest significant difference at each fertilisation rate

*, **, *** significant at *p* < 0.05, 0.01, 0.001 probability level respectively

**Fig. 2:**
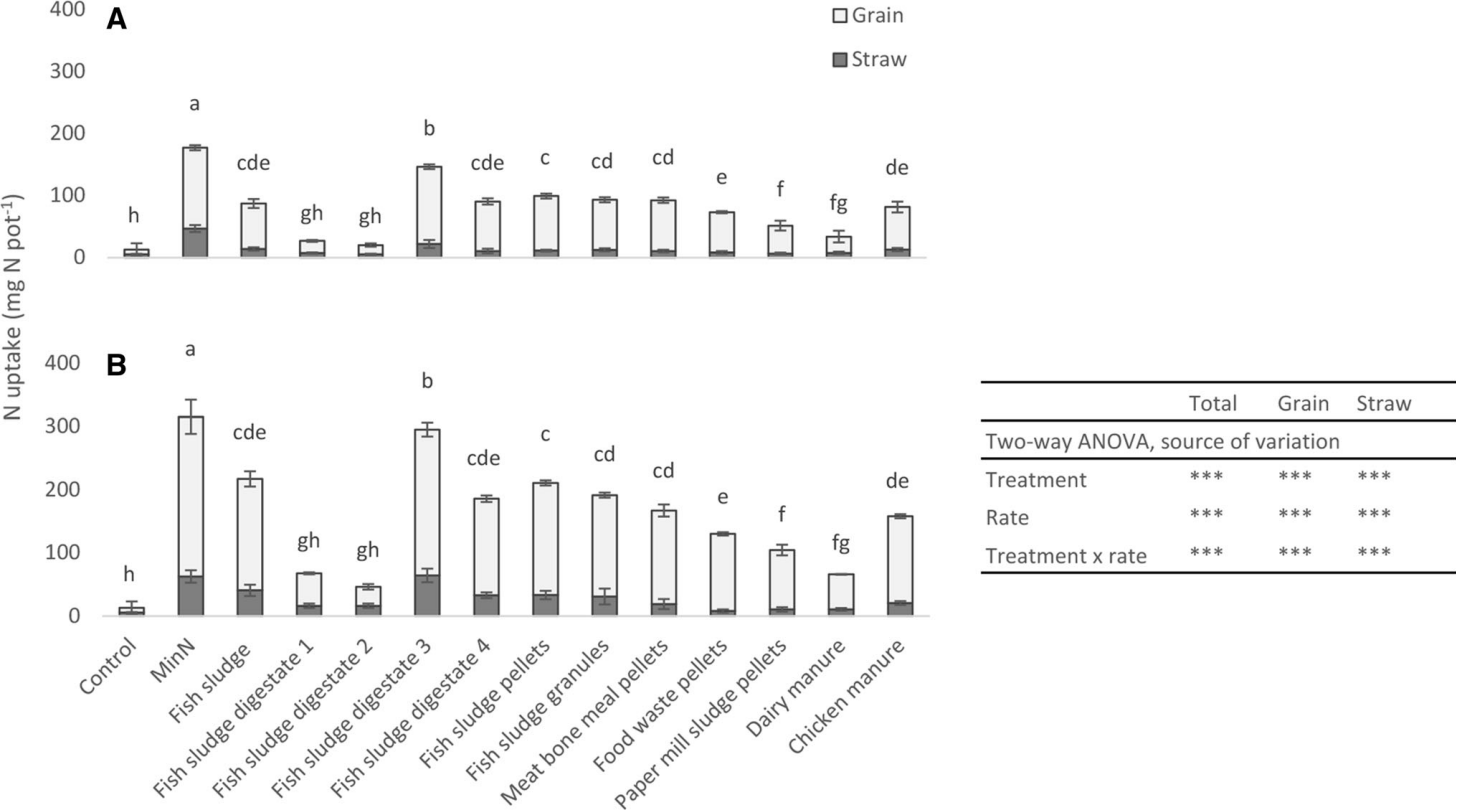
Nitrogen (N) uptake in straw and grain of barley (mg N pot^−1^) as an effect of the different fertiliser treatments at the fertilisation rates **a** 200 mg N pot^−1^ and **b** 400 mg N pot^−1^ in the bioassay. *Error bars* represent the standard deviation within each treatment. *Letters* indicate significant differences between treatments according to Tukey’s test (one-way ANOVA for each fertilisation rate)

Digestate 3 resulted in the highest N uptake in grain and straw among all recycling fertilisers at both fertilisation rates, but N uptake was lower than after application of MinN (Fig. 2) resulting in RAE of 90–101%. Untreated fish sludge, digestate 4 and both dry fish sludge products had lower N fertilisation effects than MinN and digestate 3, but resulted in equally high or higher N fertilisation effects (grain yield, N uptake and ANR) than meat-bone meal at both fertilisation rates, with RAE of 57–69% (Fig. 2; Table 3). Food waste pellets, paper mill sludge pellets and dairy manure resulted in significantly lower N fertilisation effects than untreated fish sludge, digestate 3 and 4 and the dried fish sludge products at both fertilisation rates, with RAE of 13–40%. Digestates 1 and 2 had lower N fertilisation effects than all other fish sludge products at both fertilisation rates and the effects were at the same level as those of dairy manure, on which digestates 1 and 2 were based. The N uptake in grain and straw was equally low after application of digestates 1 and 2 as after the NoN treatment, with RAE of 0–20%.

### Mineralisation of N in recycling fertilisers during the incubation experiment

The results of the incubation experiment were in agreement with the results of the bioassay, and the recovery of mineral N at day 60 of the incubation resulted in a significant linear relationship with RAE, when both fertilisation rates were included in the regression model (*p* < 0.05, *R* ^2^ = 0.71). 

**Fig. 3:**
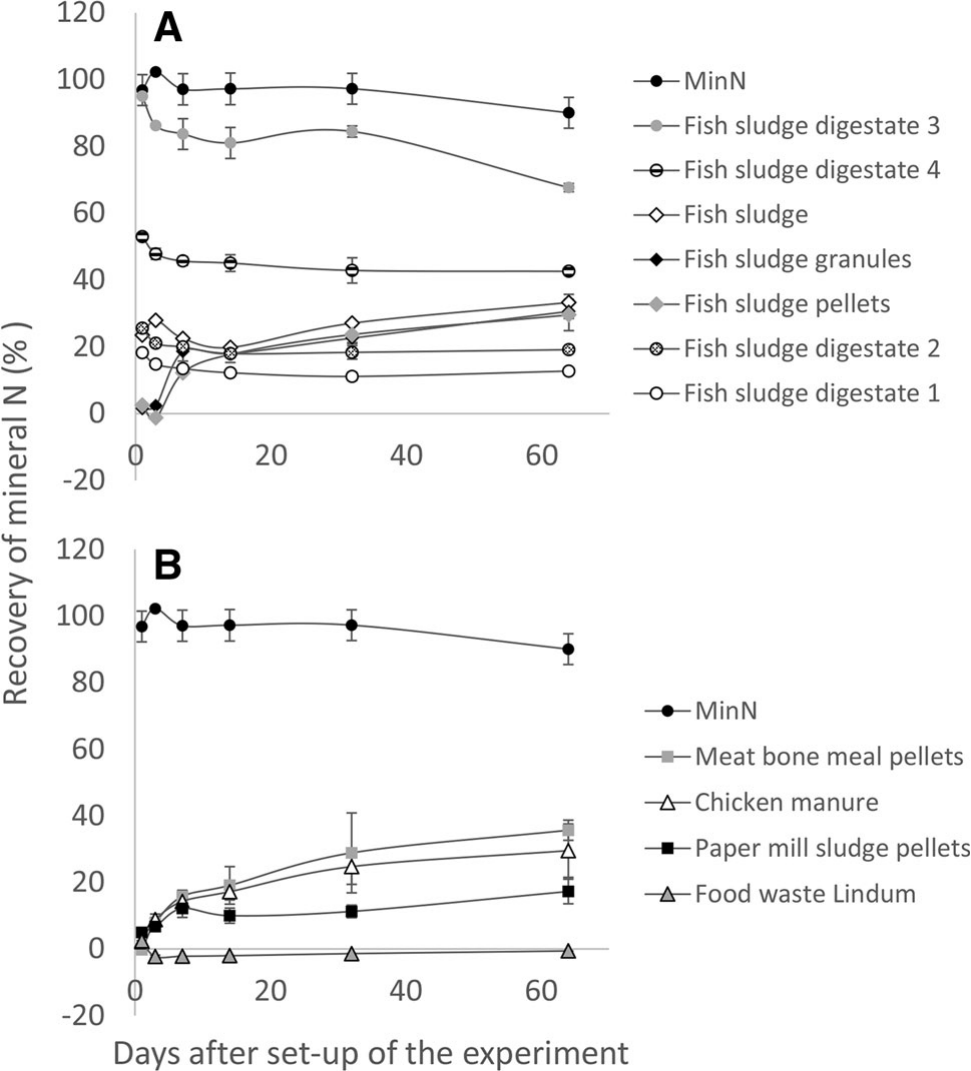
Recovery of mineral nitrogen (N) (% of total N applied) during the aerobic incubation of **a** fish sludge-based recycling fertilisers and **b** reference recycling fertilisers as a function of days after addition of fertiliser to the soil. *Error bars* represent the standard deviation within each treatment

All N applied with MinN was recovered at all time points of the incubation experiment (Fig. 3). Digestate 3 resulted in equally high recovery of mineral N as MinN at day 1 and in higher N recovery than all other recycling fertilisers at all time points, but N recovery decreased significantly throughout the experiment. Compared with digestate 3, recovery of mineral N was significantly lower after application of digestate 4 (53 ± 0.6%), digestate 1 (18 ± 0.5%) and digestate 2 (26 ± 0.5%) at day 1, and after their application N recovery also decreased significantly throughout the experiment.

In contrast, on day 1 after application of the dry fish sludge pellets and granules, recovery of mineral N was as low as 2.5 ± 0.2% and 1.5 ± 0.3%, respectively, while it was 23.5 ± 0.5% after application of untreated fish sludge. However, N recovery significantly increased throughout the experiment. The N recovery also increased for the organic reference fertilisers. However, the N in Lindum food waste did not mineralise and a significant decrease in recovery of mineral N throughout the experiment indicated immobilisation of N after this treatment.

### Fertilisation effects of recycling fertilisers in the field experiment

At Apelsvoll, in 2012 dried fish sludge did not significantly increase grain yield or N uptake in grain compared with unfertilised plants (Table 4). In contrast, meat-bone meal + digestate based on food waste resulted in equally high grain yield and N uptake in grain as mineral compound fertiliser MinNPK. In 2012, there were no significant differences in ANR in grain or RAE_field_ based on N uptake in grain between the fertiliser treatments, due to large variations. In the following year, dried fish sludge significantly increased grain yield compared with unfertilised plants and resulted in equally high N uptake in grain and ANR as MinNPK. Fish sludge resulted in RAE_field_ of 81%, which was at the same level as observed for meat-bone meal + digestate (RAE_field_ = 48%). Lindum food waste had no N fertilisation effect (RAE_field_ = 7%).

**Table 4:** Grain yields, nitrogen (N) uptake in grain, apparent nitrogen recovery (ANR) in grain and relative agronomic efficiency (RAE_field_) as an effect of different fertiliser treatments in the field experiment at two sites in 2012 and 2013

Year	Treatment	Fertilisation rate	Grain yield	N uptake in grain	ANR	RAE_field_
		kg N ha^−1^	Mg ha^−1^	kg ha^−1^	%	%
Apelsvoll (*n* = 6)						
2012	Control	0	1.8	25	–	–
	Food waste	80	2.5	38	15	39
	Fish sludge	80	2.7	41	19	52
	Meat-bone meal + digestate	80	3.2	44	24	61
	MinNPK	80	3.8	57	39	100^a^
	HSD		1.0	19	25	54
	SEM		0.2	5	6	15.0
2013	Control	0	0.9	14	–	–
	Food waste	56	1.3	19	10	30
	Fish sludge	63	2.3	31	28	81
	Meat-bone meal + digestate	80	2.0	27	17	48
	Lindum food waste	80	1.0	16	3	7
	MinNPK	80	3.1	41	35	100^a^
	HSD		0.7	12	16	38
	SEM		0.2	3	4	10
Værnes (n = 3)						
2012	Control	0	0.9	12	–	–
	Food waste	80	1.9	21	11	35
	Fish sludge	80	2.7	29	21	66
	Meat-bone meal + digestate	80	2.2	26	17	54
	MinNPK	80	3.3	38	32	100^a^
	HSD		0.2	6	8	22
	SEM		0.04	1	2	5

^a^By definition set to 100%. SEM = pooled standard error of the mean and HSD = Tukey’s honest significant difference at each fertilisation rate

At Værnes, dried fish sludge was only applied in 2012. Dried fish sludge had a significantly increasing effect on grain yield compared with the unfertilised control. It was the recycling fertiliser resulting in the highest grain yield, with RAE_field_ of 66%, even though grain yield was lower than after application of MinNPK. Nitrogen uptake in grain and ANR were also lower than after application of MinNPK, but at the same level as after application of meat-bone meal + digestate.

### Logistics

The estimated logistics costs for the two alternative solutions for the case hatchery are presented in Table 5. Yearly transportation and gate fee costs were very high for fish sludge digestate, while capital investment and running costs were the main cost drivers for dried fish sludge, which also generated some revenue. In total, dried fish sludge resulted in €160 per year higher estimated costs than fish sludge digestate. However, given the uncertainty in the parameter values, the two alternatives can be regarded as equal in terms of costs.

**Table 5:** Estimated annual logistics costs related to the treatment alternatives *fish sludge digestate* and *dried fish sludge* calculated for the case hatchery Smøla Klekkeri og Settefiskanlegg

Alternative	Fish sludge digestate	Dried fish sludge
Transportation	40 300	4440
Gate fee	59 850	–
Labour costs	–	50 000
Energy costs	–	13 000
Depreciation costs	1400	43 750
Operating costs	101 550	111 190
Finance costs	1400	17 500
Revenue from selling fertiliser		25 900
Total costs	102 950	102 790

Related costs are given in Euro (€)

## Discussion

### Nitrogen fertiliser effect of anaerobic digestate

This study showed that the N fertiliser effect of fish sludge-based recycling fertilisers can be good, but that the effect varies depending on the treatment technology applied to the fish sludge (Fig. 2; Table 3).

Both the bioassay and the incubation experiment suggested that the N fertilisation effects of digestates based on fish sludge and dairy manure are equivalent to the fraction of mineral N they contain. In digestate 3, all N was present as mineral N and accordingly digestate 3 had equally good fertilisation effect as MinN in the bioassay (Tables 2, 3). Good fertilisation effects were confirmed by equally high recovery of mineral N, as found for MinN during the incubation experiment (Fig. 3). In digestates 1 and 2, on the other hand, only <30% of total N was present as mineral N, which was reflected by equally low N uptake in grain and straw as in the unfertilised control treatment (NoN). The fact that the N fertilisation effect of anaerobic digestates is equivalent to their fraction of mineral N is known from other studies (Haraldsen et al. 2010; Möller and Müller 2012). Including four digestates based on varying ratios of fish sludge and dairy manure in this study allowed us to conclude that the higher the fraction of fish sludge in the anaerobic digestion process, the higher the fraction of mineral N in the digestate (Tables 1, 2).

Due to a methodological artefact, the amount of NH_4_-N in digestate 3 exceeded its total N content (Table 2). This inconsistency can have resulted from the determination of total N, NH_4_-N or DM for the heterogeneous waste resource. If the total N content in the digestate had been higher than assumed, more N than intended would have been applied. This means that the real fertilisation effect of digestate 3 would have been lower than concluded from the experiments.

Previous studies have shown that fish sludge has large biogas potential. However, when fish sludge is the only substrate in anaerobic digestion, accumulation of ammonia, long-chain fatty acids and volatile fatty acids can result in inhibition of the biogas process (Gebauer and Eikebrokk 2006). In practice, it will therefore be challenging to run stable anaerobic digestion processes with >20 vol% fish sludge (Gebauer et al. 2016) resulting in digestates with a similar composition to digestates 1 and 2, unless the digestion process can be adapted to tolerate larger fractions of fish sludge. Both the mineral N content and the N fertilisation effect of dairy manure were rather low (Tables 2, 3, e.g. Sørensen et al. 2003), which can contribute to explaining the poor fertiliser performance of digestates 1 and 2 with the highest ratio of dairy manure and unusually low mineral N contents (Möller and Müller 2012).

The results of both the bioassay and the incubation experiment also indicated that the organic N in digestates based on fish sludge and manure does not mineralise fast enough to supply crops with sufficient N. During anaerobic digestion of organic matter, easily degradable carbon is preferentially transformed to biogas compared with recalcitrant organic compounds (Möller 2015). Organic N remaining in the digestate will therefore probably be present in stable, recalcitrant compounds and will not result in desirable net mineralisation. In comparison to previous studies on various organic materials (e.g. Janssen 1996), here the C:N ratios of the recycling fertilisers failed to explain RAE in the bioassay (*p* = 0.82, *R* ^2^ = 0.003). Decreasing recovery of mineral N during the incubation experiment even indicated that microbes applied with the digestate might have caused immobilisation of mineral N during degradation of organic matter. Accordingly, Alburquerque et al. (2012) reported immobilisation of inorganic N during an incubation experiment with six digestates based on co-digestion of pig or cattle slurry with different agro-industrial residues using a sandy loam. Microbial immobilisation could have been promoted by the high C:N ratio in the sphagnum peat of the model soil used here. Immobilisation of mineral N was also likely to have happened after application of Lindum food waste, as indicated by the incubation experiment (Fig. 3b) and lack of N fertilisation effects during the field experiment in 2013 at Apelsvoll (Table 4).

We used a model soil with a low content of organic C in the bioassay and the incubation experiment, which was probably also characterised by low microbial activity. Therefore, the incubation experiment did not allow the total net-mineralisation potential of organic N in the recycling fertilisers to be determined. However, the incubation experiment made it possible to compare the mineralisation potential of the fertiliser products included in the study in a standardised environment. If agricultural soil with high microbial activity had been used, we might have seen higher mineralisation rates than observed, but overall the results of the bioassay and incubation experiment were in agreement with those of the field experiment.

### Nitrogen fertilisation effect of dried fish sludge

In the dried fish sludge products, N was mainly present as organic N (Table 2). The bioassay, the incubation experiment and the field experiment all indicated that organic N in dried fish sludge mineralises rapidly and quickly becomes available to plants. This is in agreement with the results of earlier pot experiments that studied the N fertilisation effect of fish sludge treated by the Global Enviro method, using ryegrass and spring cereals as experimental crops, and reporting RAE ranging from 50 to 90% (Brod et al. 2012, 2014). In the present study, fertilisation effects in the bioassay and under field conditions and the mineralisation rate of dried fish sludge products were similar to those observed for meat-bone meal, as has been documented in a range of previous studies (e.g. Jeng et al. 2004, 2006; Delin et al. 2012). Meat-bone meal is an ingredient in different fertiliser products also used in organic farming.

### Recommendations and regulations

This study showed that fish sludge can have good effects as an N fertiliser, but the nutrients in fish sludge can only be efficiently recycled if applied to agricultural land where nutrient inputs as fertiliser are needed to achieve optimal needs. Fish sludge is commonly generated along the coastline of north-west Norway and agricultural soils in western Norway are already characterised by high P levels due to high livestock density (Hanserud et al. 2016). Furthermore, Hamilton et al. (2017) have shown that the total amount of P in fish sludge exceeded the demand for P fertiliser in Norway already in 2011, and aquaculture production is anticipated to grow considerably in the future. Therefore, nutrients in fish sludge can only be efficiently utilised as fertiliser if transported to agricultural land in need of nutrients, e.g. in mainland Europe.

Transporting dried fish sludge will probably be more cost- and energy-efficient than transporting nutrients in anaerobic digestate based on fish sludge. The simple logistics analysis of a representative smolt hatchery conducted here showed that transportation of fish sludge to central biogas plants was one of the main cost drivers for the fish sludge digestate treatment alternative. Research is currently being conducted on anaerobic digestion of fish sludge as the only input substrate in a small on-site plant at the hatchery (iLaks 2016). However, the logistics analysis presented here did not consider transportation of digestate from the biogas plant to agricultural land, which is probably not economically viable unless nutrients are concentrated during post-treatment.

The European regulation regulating the use of CE-marked fertiliser products is currently under revision (EC 2016). Both dried fish sludge products studied here fulfilled the suggested requirements for solid organic fertilisers in terms of nutrient content and contaminants (Table 2), allowing them to be marketed as CE fertiliser products in Europe. Digestates 1, 2 and 3 also fulfilled the requirements for liquid organic fertilisers, whereas digestate 4 exceeded suggested cadmium limitations, set to 1.5 mg Cd kg^−1^ DM. Furthermore, our study showed that declaration of total N content together with the fraction of NH_4_-N, as suggested in regulations, can be insufficient to reflect good N fertilisation effects of recycling fertilisers containing rapidly mineralising organic N, such as dried fish sludge.

## Conclusions

The linearity of nutrient flows makes current Norwegian fish farming practices highly unsustainable. Hatcheries are meeting increasingly strict requirements to collect fish sludge before discharging water to the sea and are actively seeking cost-efficient solutions to handle the sludge. A bioassay, incubation and field experiment showed that dried fish sludge has the potential to replace 50–80% of nitrogen in mineral fertiliser, while the nitrogen fertilisation effect of anaerobic digestate increases with increasing fraction of fish sludge compared with dairy manure. This suggests that Norway can become an exporter of recycling fertilisers based on fish sludge if the quality of the fish sludge product as fertiliser is influencing decision making on how to process the sludge and if European regulations facilitate use of fish sludge as a fertiliser.

## Electronic supplementary material

Below is the link to the electronic supplementary material.

Supplementary material 1 (PDF 25 kb)
