# Supplementary material for: Drying or anaerobic digestion of fish sludge: Nitrogen fertilisation effects and logistics
Source: Ambio. 2017 Jun 7;46(8):852–64. doi: 10.1007/s13280-017-0927-5 (PMC5639799; doi:10.1007/s13280-017-0927-5)
Supplement: Supplementary file 1 — Supplementary material 1 (PDF 25 kb) [file 13280_2017_927_MOESM1_ESM.pdf]

Ambio

Electronic Supplementary Material
This supplementary material has not been peer-reviewed.

Title: **Drying or anaerobic digestion of fish sludge: Nitrogen fertilisation effects and logistics**

Chemical properties of the recycling fertilisers used in the field experiment

Tables S1 and S2 provide an overview of selected chemical properties of recycling fertilisers used in field experiments in 2012 and 2013, respectively. Parameters were determined as described, unless otherwise indicated.

Table S1. Chemical properties of recycling fertilisers used in the field experiment in 2012

		Food waste	Fish sludge	Meat-bone meal	Digestate, source-separated food waste
DM	g $100g^{-1}$	93.5	80.5	97	3.6
OM	g $100g^{-1}$ DM	83.8	82.1	65	-
pH		5.6	5.6	-	8.4
N	g kg^{-1} DM	50.3	87.0	82.5	108
NH_4-N	g kg^{-1} DM	5.1	17.4	0.44	69
Nmin	% of total N	10	20	0.5	64
P	g kg^{-1} DM	19 [a]	31 [a]	60	9.2 [a]
K	g kg^{-1} DM	6.8 [a]	1.7 [a]	1.7	47 [a]
S	g kg^{-1} DM	3.7 [a]	8.3 [a]	3.5	4.4 [a]
Ca	g kg^{-1} DM	61 [a]	55 [a]	120	55 [a]
Mg	g kg^{-1} DM	1.7 [a]	5.6 [a]	1.8	3.5 [a]
Fe	mg kg^{-1} DM	140 [a]	1000 [a]	380	1800 [a]
Cd	mg kg^{-1} DM	-	-	0.015	-
Pb	mg kg^{-1} DM	-	-	<0.31	-
Hg	mg kg^{-1} DM	-	-	<0.001	-
Ni	mg kg^{-1} DM	-	-	0.66	-
Zn	mg kg^{-1} DM	57 [a]	890 [a]	86	540 [a]
Cu	mg kg^{-1} DM	11 [a]	47 [a]	4.3	45 [a]
Cr	mg kg^{-1} DM	-	-	3.3	-

[a]Extraction in aqua regia

Table S2. Chemical properties of recycling fertilisers used in the field experiment in 2013

		Food waste	Fish sludge	Meat-bone meal	Digestate, source-separated food waste	Food waste, Lindum
DM	g $100g^{-1}$	91	65	96.1	1.4	82.1
OM	g $100g^{-1}$ DM	-	-	66.9	-	-
pH		4.9	-	6.2	7.5	8.2
N	g kg^{-1} DM	34	85	82.2	200	30.5
NH_4-N	g kg^{-1} DM	3.8	-	5.0	157	3.7
Nmin	% of total N	11	-	6	79	12
P	g kg^{-1} DM	4.7	-	73[a]	100	4.9
K	g kg^{-1} DM	7.9	-	3.9 [a]	86	9.8
S	g kg^{-1} DM	2200	-	4.3 [a]	69	1.9
Ca	g kg^{-1} DM	15	-	140 [a]	34	77
Mg	g kg^{-1} DM	-	-	2.6 [a]	-	-
Fe	mg kg^{-1} DM	64	-	-	26	1400
Zn	mg kg^{-1} DM	24	-	-	<15	88
Cu	mg kg^{-1} DM	6.3	-	-	<2	19

[a]Extraction in aqua regia
